# Complete Genome Sequence of *Arthrobacter globiformis* Bacteriophage Ren19

**DOI:** 10.17912/micropub.biology.002350

**Published:** 2026-09-28

**Authors:** Lauren A. Holt, Jazmine N. Taylor, Kera S. Ridley, Devin-Danielle V. Webb, David S.I. Davenport, He Fu

**Affiliations:** 1 Department of Chemical, Biological and Bioengineering, North Carolina A&T State University, Greensboro, NC, United States; 2 Department of Physics, North Carolina A&T State University, Greensboro, NC, United States; 3 Department of Animal Sciences, North Carolina A&T State University, Greensboro, NC, United States; 4 Department of Biology, North Carolina A&T State University, Greensboro, NC, United States

## Abstract

Bacteriophage Ren19, isolated from a soil sample collected in Greensboro, North Carolina, USA, exhibits a siphovirus morphology and infects *Arthrobacter globiformis* B-2979. Its 68,603-bp genome has a GC content of 65.4% and contains 118 predicted genes. Based on gene-content similarity, Ren19 is assigned to actinobacteriophage cluster AP2.

**Figure 1. Transmission electron micrograph and genome map of bacteriophage Ren19:** (A) Negative-stain transmission electron micrograph of Ren19 stained with 1% uranyl acetate. Ren19 has a capsid diameter of 58 nm and a tail length of 241 nm. Imaging was performed at the University of Maryland, Baltimore County. (B) Genome map of Ren19. Predicted genes are represented by boxes, with the corresponding gene numbers shown inside. Genomic coordinates are indicated by the ruler, with each tick mark representing 100 bp. The map was generated using Phamerator and the Actino_Draft_655 database (Cresawn et al., 2011).

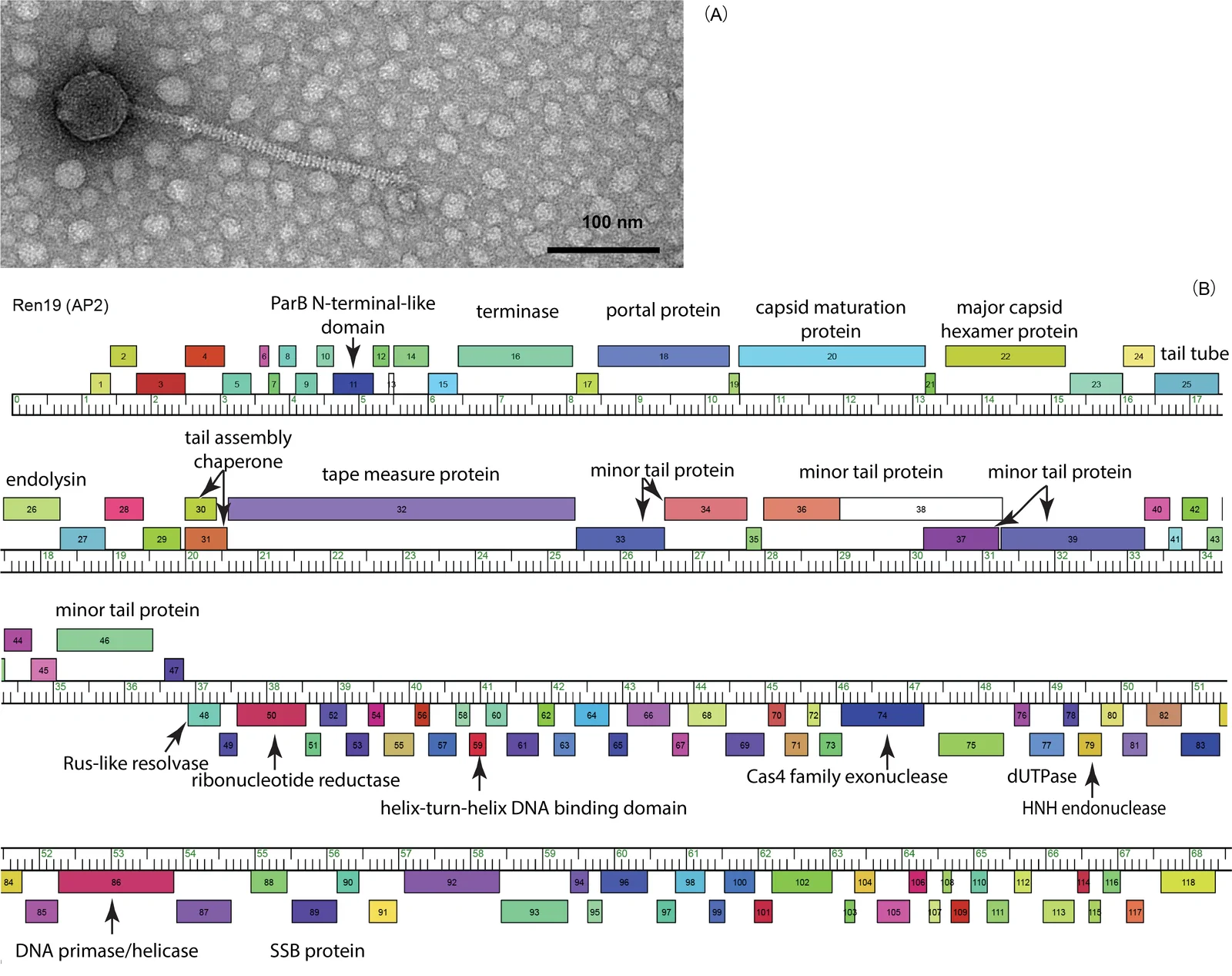



## Description

Bacteriophages are viruses that infect bacteria and constitute one of the most abundant and diverse biological entities on Earth (Hatfull, 2022). Their ability to selectively infect and kill bacterial hosts has generated growing interest in their therapeutic use against antibiotic-resistant infections (Sawa et al., 2024). Because most phages have relatively narrow host ranges, the continued isolation and characterization of novel bacteriophages are essential for expanding the repertoire of potential therapeutic candidates. To this end, we report the isolation and characterization of Ren19, a novel bacteriophage recovered from flowerbed soil on the campus of North Carolina Agricultural and Technical State University in Greensboro, North Carolina, USA (36.079194° N, 79.772028° W).

Ren19 was isolated using a modified direct-isolation procedure (Zorawik et al., 2024). Briefly, the soil sample was suspended in phage buffer by vigorous shaking for 3 h, allowed to settle overnight at 4°C, and filtered through a 0.22-µmpore-size filter. The filtrate was plated with *A. globiformis* B-2979 in PYCa top agar and incubated at 30°C for 48 h. Ren19 produced small, clear plaques (1.4 mm diameter, n = 5) and was purified through two successive rounds of plaque isolation. Negative-stain transmission electron microscopy using 1% uranyl acetate revealed a siphovirus morphology, with a capsid diameter of 58 nm and a tail length of 241 nm (n = 1; Fig. 1A ). Dimensions were measured using ImageJ version 1.54 (Schneider et al., 2012).

A lysate for Ren19 was prepared (1.5 x 1010 PFU/mL) and used to extract DNA with the Promega Wizard DNA kit. Phage DNA was then prepared for sequencing with the NEB Ultra FS kit and sequenced on an Illumina NextSeq 1000 (XLEAP-P1 kit), yielding 506k single-end 100 base reads. Raw sequencing reads were trimmed using cutadapt v4.7 (using the option: –nextseq-trim 30) and subsequently filtered with skewer v0.2.2 (using the options: -q 20 -Q 30 -n -l 50) prior to assembly. The genome was assembled using Newbler v.29 (Miller et al., 2010) and checked for completeness using Consed v2.9 (Gordon et al., 1998), resulting in an assembled genome of 68,603 base pairs with 683-fold coverage. The genome consisted of 65.4 % GC content, with a direct terminal repeat of 614 base pairs.

Glimmer v. 3.02 (Delcher et al., 1999), and Genemark v. 2.5p (Besemer & Borodovsky, 2005) were used to auto-annotate the bacteriophage genome in DNA Master (Pope & Jacobs-Sera, 2018). The auto-annotation was refined using Genemark, Phamerator (using Actino_draft database v655) (Cresawn et al., 2011), Starterator (phages.wustl.edu/starterator/), and BLAST (Sayers et al., 2025) using the Actinobacteriophage and NCBI non-redundant databases. Gene function was assigned in PECAAN (https://discover.kbrinsgd.org) and refined in PHEONA (https://www.pheona.org/) using HHPred searches against the PDB_mmCIF70, Pfam- v.36, NCBI Conserved Domains databases (Söding et al., 2005) and transmembrane domain containing proteins predicted with DeepTMHMM v. 1.0.42 (Hallgren et al., 2022). No tRNAs were found for this phage using ARAGORN (https://www.trna.se/). Default parameters were used for all software.

Based on a gene-content similarity of at least 35% to phages in the Actinobacteriophage Database (PhagesDB), Ren19 was assigned to cluster AP2 (Pope et al., 2017; Russell and Hatfull, 2016). Consistent with the genomic organization of other cluster AP2 siphoviruses, the first 47 genes are transcribed in the forward direction, whereas the remaining genes are transcribed in the reverse direction. Putative functions were assigned to 34 of the 118 predicted genes ( Figure 1B ). Ren19 encodes many proteins commonly found in bacteriophages, including a terminase, portal protein, major capsid protein, major tail protein, tape-measure protein, tail-tube protein, and minor tail protein. The genome also contains identifiable genes encoding an endolysin, HNH endonuclease, tail-assembly chaperones, and capsid-maturation protease. Two enzymes involved in nucleic acid replication or recombination—a DNA primase/helicase and a RusA-like resolvase— were also identified. Like other characterized cluster AP2 phages, Ren19 lacks identifiable integrase and repressor genes, suggesting that it is unlikely to establish lysogeny.

**Data availability.**

Annotated genome sequence can be accessed for Ren19 at GenBank with Accession Number PZ744772. Sequence reads are deposited at NCBI under SRA accession number SRX31950264 .

## References

[R1] Besemer J., Borodovsky M. (2005). GeneMark: web software for gene finding in prokaryotes, eukaryotes and viruses. Nucleic Acids Research.

[R2] Cresawn Steven G, Bogel Matt, Day Nathan, Jacobs-Sera Deborah, Hendrix Roger W, Hatfull Graham F (2011). Phamerator: a bioinformatic tool for comparative bacteriophage genomics. BMC Bioinformatics.

[R3] Delcher A. (1999). Improved microbial gene identification with GLIMMER. Nucleic Acids Research.

[R4] Gordon David, Abajian Chris, Green Phil (1998). *Consed:*
A Graphical Tool for Sequence Finishing. Genome Research.

[R5] Hallgren Jeppe, Tsirigos Konstantinos D., Pedersen Mads Damgaard, Almagro Armenteros José Juan, Marcatili Paolo, Nielsen Henrik, Krogh Anders, Winther Ole (2022). DeepTMHMM predicts alpha and beta transmembrane proteins using deep neural networks.

[R6] Hatfull Graham F. (2022). Mycobacteriophages: From Petri dish to patient. PLOS Pathogens.

[R7] Pope Welkin H., Mavrich Travis N., Garlena Rebecca A., Guerrero-Bustamante Carlos A., Jacobs-Sera Deborah, Montgomery Matthew T., Russell Daniel A., Warner Marcie H., Hatfull Graham F., Science Education Alliance-Phage Hunters Advancing Genomics and Evolutionary Science (SEA-PHAGES) (2017). Bacteriophages of
*Gordonia*
spp. Display a Spectrum of Diversity and Genetic Relationships. mBio.

[R8] Pope Welkin H., Jacobs-Sera Deborah (2017). Annotation of Bacteriophage Genome Sequences Using DNA Master: An Overview. Methods in Molecular Biology.

[R9] Russell Daniel A, Hatfull Graham F (2016). PhagesDB: the actinobacteriophage database. Bioinformatics.

[R10] Russell Daniel A. (2017). Sequencing, Assembling, and Finishing Complete Bacteriophage Genomes. Methods in Molecular Biology.

[R11] Sawa Teiji, Moriyama Kiyoshi, Kinoshita Mao (2024). Current status of bacteriophage therapy for severe bacterial infections. Journal of Intensive Care.

[R12] Sayers Eric W, Beck Jeffrey, Bolton Evan E, Brister J Rodney, Chan Jessica, Connor Ryan, Feldgarden Michael, Fine Anna M, Funk Kathryn, Hoffman Jinna, Kannan Sivakumar, Kelly Christopher, Klimke William, Kim Sunghwan, Lathrop Stacy, Marchler-Bauer Aron, Murphy Terence D, O’Sullivan Chris, Schmieder Erin, Skripchenko Yuriy, Stine Adam, Thibaud-Nissen Francoise, Wang Jiyao, Ye Jian, Zellers Erin, Schneider Valerie A, Pruitt Kim D (2024). Database resources of the National Center for Biotechnology Information in 2025. Nucleic Acids Research.

[R13] Schneider Caroline A, Rasband Wayne S, Eliceiri Kevin W (2012). NIH Image to ImageJ: 25 years of image analysis. Nature Methods.

[R14] Soding J., Biegert A., Lupas A. N. (2005). The HHpred interactive server for protein homology detection and structure prediction. Nucleic Acids Research.

[R15] Zorawik Michelle, Jacobs-Sera Deborah, Freise Amanda C., Reddi Krisanavane, SEA-PHAGES (2024). Isolation of Bacteriophages on Actinobacteria Hosts. Methods in Molecular Biology.

